# Genetic Diversity and Population Structure of Nine Local Sheep Populations Bred in the Carpathia Area of Central Europe Revealed by Microsatellite Analysis

**DOI:** 10.3390/ani15162400

**Published:** 2025-08-15

**Authors:** Zuzana Sztankoová, Michal Milerski, Luboš Vostrý, Jana Rychtářová

**Affiliations:** 1 Department of Genetics and Breeding of Farm Animals, Institute of Animal Science, 104 00 Prague, Czech Republic; milerski.michal@vuzv.cz (M.M.); vostry.lubos@vuzv.cz (L.V.); 2 Department Biology of Reproduction; Institute of Animal Science, 104 00 Prague, Czech Republic; rychtarova.jana@vuzv.cz

**Keywords:** genetic variability, local sheep breeds, microsatellite marker, population structure

## Abstract

**Simple Summary:**

Local sheep breeds and the degree of genetic diversity are important for the development of conservation based on molecular characterization. In the present study, we evaluated the genetic diversity and population structure of nine sheep breeds: Czech Wallachian sheep (CVA), Sumava sheep (SUM), Improved Wallachian (IVA), Slovak Wallachian (SVA), Świniarka (SWI), Uhruska sheep (UHR), Polish Mountain sheep (POG), Ukraine sheep (UKR), and Tsurcana (TUR) bred in Carpathia region (Czech Republic, Slovakia, Poland, Ukraine, Romania) using a set of 13 microsatellite loci. Our results showed two main gene pools. CVA and SWI were assigned to a separate gene pool, and the second pool consisted of IVA, SUM, SVA, POG, UHR, UK, and TUR, with a high level of overlap, indicating that these breeds share a common ancestor and are not clearly differentiated. The selected set of microsatellite loci can further be used for the analysis of genetic diversity.

**Abstract:**

A necessary step towards the development of genetic diversity is the protection of the valuable genetic resources of farm animals that are at risk of extinction. We analyzed 375 individuals of nine local sheep breeds bred in Central Europe (Carpathia area) from Czech Republic, Slovakia, Poland, Ukraine, and Romania using a panel of 13 microsatellite markers to investigate genetic differences and evaluate the genetic structure among and within breeds, thereby improving future breeding and conservation strategies. The mean number of alleles was 8.84, the mean number of effective alleles was 4.76, and the polymorphism information content (PIC) was 0.79. Diversity was measured using principal coordinate analysis (PCoA) as well as genetic structure, which revealed two main clusters. The first cluster was the Czech Wallachian sheep (CVA) and the Świniarka (SWI). The second cluster consisted the Improved Wallachian sheep (IVA), the Šumava sheep (SUM), the Slovak Wallachian sheep (SVA), the Polish Mountain sheep (POG), the Uhruska sheep (UHR), the Ukrainian sheep (UKR) and the Tsurcana sheep (TUR). The values of genetic distance and the fixation coefficient indicate sufficient differences between the analyzed breeds (Gst = 0.052 and Fst = 0.063). Negative values of the inbreeding coefficient also confirmed the predominance of outbreeding (Fis = −0.015). The results obtained may be helpful in breeding programs and conservation plans for local sheep breeds, as their genetic resources must be preserved to maintain an adequate level of biodiversity in animal husbandry.

## 1. Introduction

Globally, sheep have played an essential role in the socio-economic lives of people since the Neolithic period, approximately 9000 years ago, when they were domesticated from at least three ancestral subspecies of the wild mouflon (*Ovis gmelini*) [1,2]. Along with the goat, sheep are among the oldest domesticated animals on this planet. Sheep breeding in the present-day Czech Republic dates back to the ninth century. Its breeding is associated with Slavic settlement, according to historical sources, where the Wallachian-mountainous breeding method was developed in the Carpathians, Beskydy Mountains, Šumava, and the southern region of Poland [3,4,5]. These sheep had coarse wool. Sheep are primarily used as a relatively important element of traditional agricultural production for grazing grass in foothills and mountainous areas. At the same time, they are bred for milk and meat production, and the production of wool and its subsequent processing and use in the agricultural sector, the chemical industry, and construction [4,5].

Microsatellite markers (MMs) are extensively used to evaluate genetic variability and estimate the genetic distances of indigenous breeds or among closely related populations, and it has been proven in numerous studies [1,2,6,7,8,9,10,11,12,13,14] that the knowledge of genetic variation may be directly applicable in a selective breeding program. Therefore, MMs and SNPs are the most popular markers recommended by the Food and Agriculture Organization (FAO) https://www.fao.org/dad-is, (https://openknowledge.fao.org/handle/20.500.14283/i2413e) (accessed on 12 March 2025) for livestock genetic characterization studies; they are deemed one of the most valuable genetic markers for genetic characterization.

Genetic diversity is one of the key components of biodiversity, alongside ecosystems and species, and it is essential for populations or species to evolve in the presence of changing climatic or environmental conditions, as well as for increasing the fitness of animals in existing ecological conditions [15]. Thus, to maintain genetic diversity, it is necessary to control inbreeding and common origin, as stated by Odjakova et al. [10] and Olschewski and Hinrichs [16]. However, in recent decades, local breeding has been declining (107 extinct breeds of sheep), caused by the spread of commercial breeds with specialized production, the overuse of a small number of rams (increased by artificial insemination), and modern selection procedures [4,8,16,17].

As noted by Gurgul et al. [3], despite the existence of several studies on genetic variation and selection signatures in sheep, information on the genetic background and variability of local sheep populations, particularly at the genome-wide level, remains lacking. The existence of a large number of local populations with visible phenotypic differentiation encourages researchers to conduct further studies analyzing genetic differentiation in indigenous breeds or rare local populations [4,11,18,19]. As Meyermans et al. [20] describe, local sheep breeds often play an essential role in cultural heritage, can play a key role in local economies, have unique characteristics adapted to local climates, and can be invaluable in sustainable and resilient agricultural systems (maintaining landscapes and biodiversity) (FAO) [21].

The aims of this study were: (i) to compare and characterize the genetic variability and structure of nine local sheep breeds assessed using microsatellite loci recommended by FAO, and (ii) to assess and compare the possible impact of genetic admixture with other breeds bred in the Carpathian region.

## 2. Materials and Methods

### 2.1. DNA Isolation

Genomic DNA was isolated from 375 hair samples from randomly selected individuals of 9 sheep breeds beep in the Czech Republic, Slovakia, Poland, Romania, and Ukraine using a NucleoSpin Tissue kit (Macherey-Nagel, Düren, Germany, iBiotech.cz Ltd., Prague, Czech Republic) according to the manufacturer’s instruction. To determine the quality and quantity of DNA samples, we used a NanoDropTM 1000 (Spectrophotometer, Thermo Fisher Scientific, Waltham, MA, USA) with 3% agarose gel electrophoresis, and the result were then visualized on UV transilluminator gel documentation systems (G-box, Trigon Ltd., Prague, Czech Republic) after staining with Ethidium Bromide (EtBr) (Top-Bio Ltd., Vestec, Czech Republic). The isolated DNA was stored at −20 °C before analysis.

### 2.2. Microsatellite Markers

The set of 13 microsatellite markers ISRCRSP9, MAF65, MCM527, CSRD247, ILSTS11, SRCRSP23, SPS113, TGLA53, INRA23, OarFCB20, SRCRSP5, INRA063, and SRCRSP8) was used to genotype 375 animals belonging to nine sheep breeds: Czech Wallachian sheep (CVA, 35), Improved Wallachian (IVA, 59), Sumava sheep (SUM, 56), Slovakian population: Slovak Wallachian (SVA, 56), Polish population: Polish Mountain sheep (POG, 47), Świniarka (SWI, 34), Uhruska (UHR, 19), Ukrainian population: Ukraine sheep (UKR, 19) and Romanian sheep population: Tsurcana (TUR, 60). All loci were chosen based on their level of allelic diversity and location on different chromosomes and selected according to the recommendations of the International Society for Animal Genetics (ISAG) (https://www.Isag.org.uk/Docs/2005_panelsMarkersSheepGoats.Pdf) and (FAO) (https://www.fao.org/dad-is/) (accessed on 12 March 2025).

### 2.3. PCR and Fragmentation Analysis

According to the annealing temperature, all samples were analyzed in three multiplex groups (Supplementary Table S1). Multiplex polymerase chain reaction (PCR) amplifications were carried out in a 10 µL total reaction volume, containing 5 µL of Master mix 2x MyTaq^TM^ HS Mix (Bioline. Ltd., Prague, Czech Republic). We used 0.2–0.45 µL primers (10 pmol/µL; 0.01 mM of each with the forward labelled with 6-FAM, VIC, PET and NED), ~50 ng DNA and sterile water up to volume. The PCR was carried out in a standard gradient thermocycler (Biometra—T Gradient, IBiotech, Prague, Czech Republic) according to the following program: initial denaturation at a temperature of 95 °C for 15 min, followed by 32 cycles with the following steps: 94 °C for 30 s, 55 °C for 90 s, 72 °C for 1 min, and, finally, an elongation step at 72 °C for 30 min.

The obtained PCR product (1 µL) was subsequently transferred to a PCR plate together with 10 µL of Formamide (Applied Biosystems, Foster City, CA, USA), 0.5 µL of size standard LIZ500 (Thermo Fisher Scientific, Waltham, MA, USA) and 0.5 µL of redistilled water, added to a total reaction volume of 11 µL. This multiplex PCR/LIZ/Formamide mixture was incubated at 95 °C for 5 min and cooled at 4 °C in a standard gradient thermocycler (Biometra—T Gradient, IBiotech, Prague, Czech Republic) and then immediately cooled on ice for 3 min before performing capillary electrophoresis on an ABI PRISM3130 Genetic Analyzer (Applied Biosystems, Foster City, CA, USA) equipped with 36 cm long capillaries. GeneScan 500LIZ dye Size Standard (Thermo Fisher Scientific, Waltham, MA, USA) was used as a ladder. GeneMapperTM software v. 4.0 (Applied Biosystems, Foster City, CA, USA) was used for data analysis and evaluation.

### 2.4. General Statistical Analysis

We determined the number of different alleles (Na), number of effective alleles (Ne), the number of private alleles (PA), the Shannon’s Information Index (I), observed heterozygosity (Ho) and expected heterozygosity (He), the number of effective migrants (Nm), and fixation indices (Fis, Fit and Fst). We used the Hardy–Weinberg equilibrium test per locus across breeds, and markers, principal coordinate analysis (PCoA), and analysis of molecular variance (AMOVA) using the calculated genetic distance among the nine studied central European sheep breeds to evaluate the genetic differentiation among all flocks; the values were calculated using GeneAIEx v. 6.5 [22]. The polymorphic information content (PIC) of each locus was calculated using the equation of Botstein et al. [23], Power Marker v 3.0. [24]. The following genetic distance measures were calculated (Power Marker v 3.0.) to estimate the similarity between breeds: standard genetic distance DS according to Nei (1972, DS) [25] and Rogers (1972, DR) [26], with distance DC as defined by Cavalli-Sforza (1967) [27] and Edwards Reynolds (1983) [28]. A phylogenetic tree was constructed in PHYLIP using a neighbor-joining dendrogram based on the method of Saitou and Nei [29]. STRUCTURE software version 2.3.4 [30] was used for the analysis of genetic structure; individuals were assigned to clusters using the Bayesian method under an admixture model. The simulation was run 10 times for each value of K (1–10), where K was the number of tested clusters. The software package Clumpak (http://clumpak.tau.ac.il/) (accessed on 12 March 2025) was utilized to identify the most probable values of K [31]. All runs were performed with a length of 100,000, followed by 150,000 Markov chain Monte Carlo (MCMC) repeats after burn-in with 20 replicate runs for each K. The estimate of the best K was calculated using the delta K method by Evanno et al. [32] and the Puechmaille statistics [33], as implemented in the STRUCTURE SELECTOR web interface [34].

## 3. Results

### 3.1. Genetic Variability of Microsatellite Markers

In the present study, all microsatellite markers were successfully amplified in all breeds, a testament to the robustness of our methodology. Furthermore, all microsatellite markers were found to be polymorphic, reinforcing the reliability of our research. The characteristics of the analyzed microsatellite markers, along with the genetic variability statistics, are detailed in Table 1, providing a comprehensive overview of our findings.

A total of 188 alleles (K) were detected in 13 analyzed microsatellite loci, with an average of 14.54 alleles per locus, ranging from 8 (SRCSP5) to 22 (CSRD247). The number of alleles (Na) per locus ranged from 5.22 (SRCSP5) to 12.67 in the SRCSP23 locus, with an average of 8.84 alleles per marker (Table 1). The average of effective alleles (Ne) was 4.76, indicating interbreed genetic diversity, with the lowest value recorded for the SRCSP5 marker (2.84) and the highest value for SRCSP23 (7.78). In our study, the average of polymorphic information content (PIC) across all loci was 0.79, which is more than 0.5, with the highest value recorded for the SRCSP23 locus (0.91) and the lowest value of 0.68 was recorded for the two markers SRCSP5 and SRCSP9, which indicates that all loci were highly polymorphic. The He value was balanced with the Ho value, indicating balanced heterozygosity. The highest mean Ho value was reported at the ILSTS11 locus (0.837) and the lowest at the SRCSP5 locus (0.48), indicating a high level of genetic variability among the nine breeds. The mean Ho value was 0.765, and the value for He (0.753) was similar. Similarly, the mean value of the total expected heterozygosity (Ht) was 0.804. However, the lowest value was found at the SRCSP5 locus (0.680), while the highest value was recorded at the SRCSP23 locus (0.913), representing the most significant genetic variability.

F-statistics were calculated, which analyze the degree of differentiation within and between populations, and the inbreeding coefficient (Fis) varied from −1 to +1 [35]. The highest level of heterozygous deficiency (Fis and Fit) within the population was observed for the locus SRCSP5 (0.244 and 0.294, respectively), while the lowest levels were observed in the locus SRCSP9 (Fis = −0.162 and −0.105). The other loci were close to zero or below zero. The Fis across the analyzed markers showed a negative value of −1.5%, indicating an absence of inbreeding in the population. The global deficit of heterozygotes (Fit) was approximately 5% across the population. To measure the degree of genetic variability between breeds, we used the mean Fst index. The multilocus Fst analysis revealed that only 6.3% of the total genetic variation in the examined breeds is between breeds, while the remaining 93.7% is attributed to individual differences. Shannon’s informative index ranged from 1.241 for SRCSP5 to 2.210 for the SRCSP23 locus, representing an average value of 1.701, which can be characterized as a high degree of overall genetic variability; all loci were very informative, making them very useful for genetic diversity studies in the investigated population which measures the biodiversity level in a population. Mean Gst and Gis, analogs of Fst and Fis, indicate sufficient differences between the analyzed breeds and at the same time, they point to the prevailing outbreeding in the population with a value of −0.1%, indicating an inbreeding coefficient within subpopulations and individuals, respectively (Table 1).

Mean Gst and Gis, analogs of Fst and Fis, were 5.2% of the total genetic diversity portioned among breeds, and 94.8% was within populations, with a value of −0.1%, indicating an inbreeding coefficient within subpopulations and individuals, respectively (Table 1).

### 3.2. Genetic Variability Among Populations

Table 2 and Supplementary Table S2 describe results of the genetic diversity analysis among populations.

Results show that the mean number of alleles per locus in the breeds ranged from 6538 (UHR) to 11,385 (TUR), with a mean of 8838. The mean number of effective alleles was 4.756 and ranged from 3.158 (SWI) to 5.916 (IVA). The observed heterozygosity (Ho) varied from 0.71 (SWI) to 0.817 (IVA), while the expected (He) ranged from 0.649 (SWI) to 0.795 (IVA and TUR). Except for SUM, UKR, and TUR, the average He value exceeded Ho compared to the other studied breeds. The indicator of genetic diversity in the population is Shannon’s Information Index (I), which ranges from 1.308 (SWI) to 1.91 (IVA and TUR). The average value of I for the entire population was 1.701, indicating that entropies increasingly favored the most abundant alleles. The coefficient of inbreeding (Fis) varied from −0.092 (SWI) to 0.031 (TUR). In all cases, Fis was lower than 0.05, and according to the FAO, breeds are considered vulnerable when ΔF ranges from 0.5 to 1% [12]. In general, all nine sheep populations showed high genetic diversity for all loci analyzed in the current study. In all breeds, except CVA and UKR, a total of 24 private alleles were observed at 13 loci and were distributed across the nine sheep populations. Tsurcana (TUR) sheep had the highest number of private alleles (8), while the lowest number of private alleles (one) was found in the Sumava sheep (SUM) and Uhruska (UHR) sheep breeds. Hardy-Weinberg equilibrium testing was also conducted to assess the significance of inbreeding occurring at the overall loci in each population. Hardy–Weinberg equilibrium tests for all nine breeds form 13 microsatellite markers showed low deviations (*** *p* > 0.001) in only 13 of 117 HWE tests performed (Supplementary Table S2), which is due to the absence of heterozygote deficit in each breed; this deficit may be attributed to many factors, including inbreeding and selection.

Analysis of molecular variance (AMOVA), a method for determining population differentiation using molecular data, was used to detect genetic variation between individuals and populations (Supplementary Table S3). Estimation of genetic diversity within and between populations is fundamental for designing appropriate breeding and conservation programs. The obtained results revealed that there was only a 5% variation among the populations, and 94% of the total variance was found within individuals. In comparison, 1% of the variance was found among individuals within populations.

### 3.3. Genetic Distance Among Populations

The results of the pairwise differentiation coefficient (Fst) were used as a measure of genetic variation between pairs of populations and are presented in Table 3. The number of migrants (Nm) between populations ranged from 3.127 (SWI and UHR) and 21.854 (TUR and IVA), respectively, with an average of 3.857. The SWI breed showed the highest values of pairwise Fst and the lowest values of Nm compared to other breeds, indicating that this breed has maintained a significant genetic isolation from all other breeds. The highest Fst values were observed between SWI and UKR (0.076), SWI and UHR (0.074), SWI and CVA (0.068), and SWI and POG (0.065). The lowest Fst values were found between TUR and IVA (0.011) and TUR and POG (0.012). The number of migrants (Nm) for the observed populations was 21.854 and 3.127. Based on the first results, we can conclude that there is very little differentiation between the IVA and “TUR” breeds, as well as between the POG and TUR breeds compared to the SWI and UHR breeds, as well as SWI and UKR, where the greatest differentiation was recorded. As for the Czech breeds, the results show that SUM and IVA are the closest breeds; little differentiation was also recorded for the IVA and SVA breeds compared to the original CVA.

**Figure 1:**
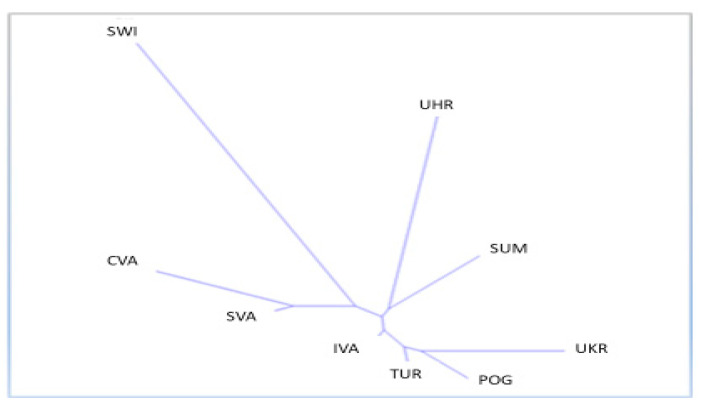
(Radial) Neighbor-joining dendrogram generated from the Reynolds genetic distances of the examined breeds. The populations are: Czech Wallachian sheep (CVA), Improved Wallachian (IVA), Sumava sheep (SUM), Slovakian population Slovak Wallachian sheep (SVA), Polish population: Polish Mountain sheep (POG), Świniarka (SWI), Uhruska (UHR), Ukrainian population: Ukraine sheep (UKR) and Romanian sheep population: Tsurcana (TUR).

Table 4 and Table 5 present the distances between the observed populations, which were quantified by the values of DS, DR, and DC and determined by the genetic distance according to [28]. In all cases, it was confirmed that the highest value of the genetic distance was between SWI and UHR, as well as between SWI and UKR, indicating that these breeds are genetically distant from each other. Conversely, the lowest value was calculated between TUR and IVA, as well as between TUR and POG, which suggests that these breeds are closely related. The results are, therefore, consistent with the calculation of the pairwise differentiation coefficient (Fst) and confirmed by principal coordinate analysis (PCoA) and the dendrogram description (Figure 1).

### 3.4. Genetic Structure and Admixture Analysis

The Reynolds genetic distances [28] were used to reconstruct a radial neighbor-joining dendrogram (Figure 1), which shows three clusters. The first cluster consisted of the breeds CVA, SVA, and SWI. The results show that the SWI breed had medium values, indicating that the given breed stood out from the other monitored breeds but formed a cluster together with the CVA and SVA breeds. The second cluster is represented by the SUM and UHR breeds, and the third cluster contains the IVA, TUR, POG, and UKR breeds. In all four cases (genetic distances), it was confirmed that the lowest distance between breeds was recorded for the TUR and IVA breeds, followed by TUR and POG, and the most significant distance was shown for the SWI and UKR breeds, followed by SWI and UHR (Table 4 and Table 5).

**Figure 2:**
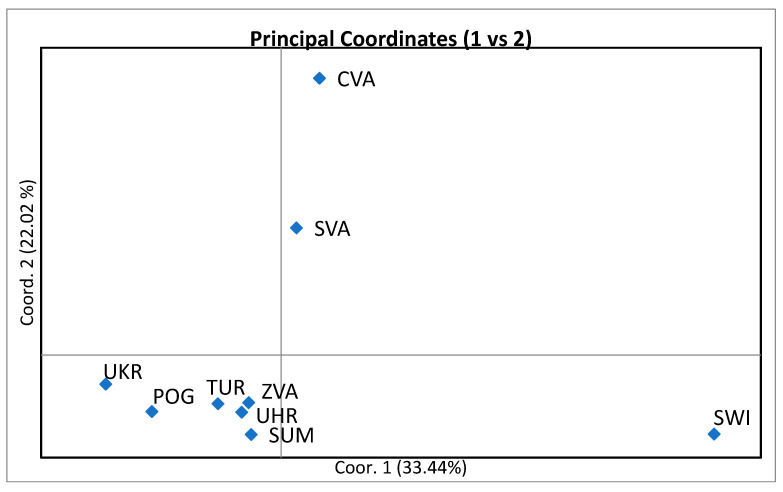
A two-dimensional plot of the principal coordinates analysis (PCoA) obtained by GenAIEX with data standardization shows the clustering of 13 microsatellite markers typed of the nine sheep breeds. The first three principal components explained 74.76% of the total variance. Czech Wallachian sheep (CVA), Improved Wallachian (IVA), Sumava sheep (SUM), Slovakian population: Slovak Wallachian sheep (SVA), Polish population: Polish Mountain sheep (POG), Świniarka (SWI), Uhruska (UHR), Ukrainian population: Ukraine sheep (UKR) and Romanian sheep population: Tsurcana (TUR).

Principal coordinate analysis (PCoA) and genetic structure analysis were performed to study genetic differentiation and admixture among nine Central European breeds. The results of principal coordinate analysis (PCoA) are graphically represented in Figure 2, where three main clusters are observed. The results are consistent with the variation revealed by the genetic distance matrix analysis described above. The first two principal components explained 54.46% of the total variance, specifically 33.44% for the first component and 22.02% for the second. The first component separated the CVA and SVA breeds, which show a very close resemblance, and they differ from SWI as well as from other populations. The SWI population is located in the second quadrant. It stands out as a separate breed compared to the different breeds, UHR-IVA-SUM-POG-UKR, which are located in the third quadrant and exhibit moderate to high homogeneity (Figure 2).

**Figure 3:**
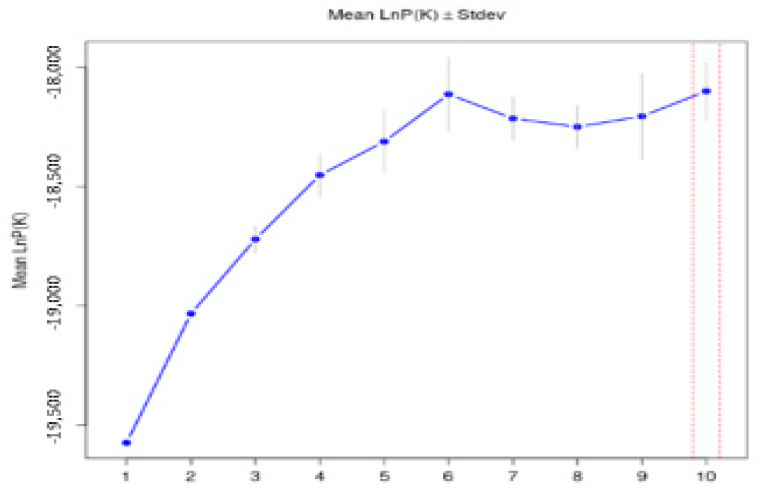
Graphical determination of the best number of clusters from structure analysis for microsatellite loci in sheep populations using the delta K method proposed by Evanno et al. [32].

Bayesian clustering analysis was conducted using the STRUCTURE software to investigate breed differentiation. According to the STRUCTURE analysis, it was assumed that the most likely number of local populations contributing to the observed genetic variability was six (k = 6), indicating that the nine local breeds analyzed in this study can be assigned to two main clusters (Figure 3, Supplementary Table S4).

**Figure 4:**
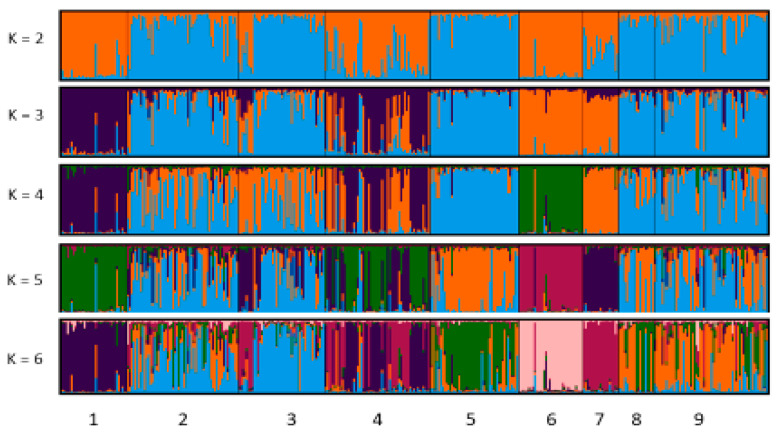
Bar plot from inferred population structure using the STRUCTURE program, based on the Bayesian grouping admixture model (K = 2 and 7); Abbreviations: Czech sheep populations: 1—CVA, Czech Wallachian sheep; 2—IVA, Improved Wallachian; 3—SUM, Sumava sheep; Slovakian population: 4—SVA, Slovak Wallachian sheep; Polish population: 5—POG, Polish Mountain sheep; 6—SWI, Świniarka; 7—UHR, Uhruska; Ukrainian population 8—UKR, Ukraine sheep; Romanian sheep population 9—TUR, Tsurcana.

The genetic population was determined based on the admixture level of each sheep. Each analyzed individual was represented by a single vertical line broken into colored segments. At K = 2, the first cluster was made up of CVA, SVA, and SWI sheep breeds, and a second group of clusters consisted of IVA, SUM, POG, UHR, UKR, and TUR. At K = 3, breeds SWI and UHR were separated from CVA and SVA; however, at a value of K = 4, CVA, POG, SWI, and UHR were identified, while the remaining populations (IVA, SUM, SVA, UKR, and TUR) showed some admixture: a high level of overlapping within two common ancestors and no difference. The six separate clusters were identified based on K = 6 (Figure 4). The clustering results were highly consistent with the phylogenetic tree, as well as with analyses of Fst and PCA.

## 4. Discussion

### 4.1. Population Genetic Diversity Based on Microsatellite Markers

Although indigenous breeds, due to their lower production, represent a high cultural value for each country and are more often replaced by commercial breeds, in order to avoid losses in productivity and to increase the economics of farming, urgent measures are required for their survival and protection. As a result, the genetic structure of indigenous sheep can be affected by demographic events, such as gene flow between different breeds and subsequent genetic mixing between them [11]. The study of gene variance plays a crucial role in the development of rational breeding programs for both economically essential animal species and animal genetic resources. This study aimed to compare and evaluate the genetic variation and relationships among nine sheep breed populations in the Carpathian area using microsatellite markers.

All markers were highly polymorphic and could be used to sufficiently evaluate the genetic diversity of sheep, that they had more than five alleles per locus. The PIC value for each locus was higher than 0.5, which represented a high degree of polymorphism, regardless of the number of selected microsatellite markers and independently of the number of monitored breeds [12,19,36,37,38,39,40].

The level of variation given by the number of alleles at each locus serves as a measure of the impact of each of the studied breeds on differentiation within livestock populations. In our research, we found that the average number of alleles per locus was 14.54 (Table 1), representing moderate to high allelic diversity across the entire sheep population. The mean number of alleles (Na) of the studied sheep population was similar to those reported for Bulgarian sheep breeds [12,38], Romanian sheep [18], and Greek sheep [41], and in an extensive study conducted by Peter et al. [6] that included 57 breeds from 15 European countries. However, a high value for the mean number of alleles per locus (20.71 per locus) was obtained in Turkish sheep breeds [40], and in the Tsigai- and Zackel-type group sheep breeds from central, eastern, and southern European regions [42]. A low value was obtained in sheep from Bosnia, Herzegovina and Croatia [43]. These findings suggest that these microsatellite markers can be reliably used to measure genetic diversity in the observed breeds.

The level of heterozygosity ranges from −1 to 1 and is expressed using the F-statistic, but the value in our study for most loci had a negative mean value of −0.015 (Table 1). This interprets a higher proportion of heterozygous individuals, which could be explained by high gene flow between breed herds and mating-related avoidance of animals. The observed Fis value was consistent with studies such as Marković et al. [44] (−0.07) and Mihailova [38] (−0.02), but lower than the values previously reported by Mihailova et al. for Bulgarian sheep [12] (0.034) and Kusza et al. [19] (0.288) Kusza et al. [42] (0.264), Polish sheep [3] (range 0.042 to 0.161), Romanian [18] (0.161) and Turkish sheep breeds [40] (0.137). Therefore, it is evident that the investigated populations exhibited heterozygosity deficiencies. Additionally, the results suggest that genetic variation, as measured by microsatellite markers, exhibits a low level of genetic differentiation between the studied breeds, which may be attributed to their shared history, indicating that they are closely related genetically.

The relatively equal values of Ho (0.764) and He (0.753) indicate, on the one hand, high diversity in the studied sheep population, but on the other hand, they also indicate the presence of inbreeding in the population, which may be caused by the preservation of local breeds. Similar results were obtained for the group of local breeds from Bulgarian sheep [11] and Montenegrin local sheep [44]. The high values of Ho and the negative value of Fis in our study indicate that individuals in the population were less closely related, as described by Odjakova et al. [11] and Mihailova [38].

Although Gst is analogous to Fst, it may be concluded that the genetic difference between the studied breeds is not substantial enough due to geographic proximity, environmental similarity, and breeding practices but is rather caused by past and current gene flow among them. The Gst value indicates that differences between individuals can explain 94.8%, while 5.2% of the total genetic variation results from differences between populations. The average Gst value was lower than that recorded for Turkish breeds (0.079) [40], Montenegrin breeds (0.072) [44] but higher than that recorded for Bulgarian breeds (0.008 and 0.018) [10,11] and Romanian breeds (0.027) [18].

The Shannon’s Information Index (I) was used to evaluate the informativeness of surveyed loci and breeds. The index across loci showed informativeness at a level of 1.701, which was comparable to that reported by Odjakova et al. [11] and Machova et al. [45]. Generally, it reflects the level of effectiveness of genetic markers and genetic diversity within the population [46].

The high polymorphic information content (PIC) values and the high average number of alleles per locus indicate that the panel of 13 microsatellite molecular markers used in this study is suitable for investigating genetic diversity. These results suggest that the microsatellite markers employed in this study, in conjunction with the latest statistical methods, provide a valuable tool for the conservation and management of endangered breeds. They have a high level of confidence in revealing the genetic diversity of these breeds. The differences between previous literature and this study were primarily due to non-comparative aspects, such as the microsatellite studied and breed-specific differences.

### 4.2. Population Genetic Diversity Among Breeds

Several indicators of variability within a breed, such as Na, Ne, and MNA, are described in Table 2. Regarding breeds, the mean number of observed alleles (MNA) ranged from 6.53 (UHR) to 11.385 (TUR). The total heterozygosity was balanced, and, in general, the observed breeds showed a high level of genetic diversity. The overall heterozygosity was balanced and generally showed a high level of genetic diversity in all breeds studied. This may indicate an isolated braking effect, possibly caused by the small and isolated population and/or by the need to cross to improve production capacity, as reported in the study conducted by Marković et al. [44]. However, the observed heterozygosity was low compared to the data presented by Mastranestasis et al. [47] in Greek sheep and higher than in Colombian sheep [48] (0.63–0.74), Turkish sheep [40] (0.57–0.74), Bulgarian sheep [19] (0.458–0.577), Croatian and Slovenian sheep (0.64–0.69) [49], and Romanian sheep [18] (0.65–0.74) However, similar results of balanced heterozygosity were reported in Montenegri sheep (0.69–0.763) by Marković et al. [44].

The H–W equilibrium test is used to determine whether a population is in equilibrium based on genotype or is deviating from equilibrium. Factors that cause imbalance include selection, mutations, genetic drift, heterozygosity deficit, small population size, and female-to-male ratio [38,39,48].

Genetic distances between observed populations describe a relatively low level of genetic differentiation, ranging from 0.011 to 0.076. Thus, only 1–7.5% of genetic variation can be attributed to differentiation by crossbreeding, while the remaining 92.5–98.5% is due to interindividual variation. Consistent results have been reported in studies [3,8,20,41,49], where the genetic differentiation of local breeds is lower than that of sheep of distant geographical origin; however, all observed populations reflect their common origin or admixture. Although we recorded an average negative value for Fis, a positive value was recorded in the breeds: (SUM, POG, UKR TUR), where the reason could be the excess of homozygotes, which is also the reason for the observed deviation from HWE. Meanwhile, in the breeds CVA, IVA, SVA, and SWI UHR, a negative value was recorded and the heterozygosity was higher. As noted by Mihailova et al. [12], the lack of clear distinction between the observed breeds may be due to the origin or migration but also to the geographical proximity and similarity of the environment. However, breeding practices are most likely based to a greater extent on only a few phenotypic characteristics typical of the breed, and these breeds we observed belong to the Zakel type breed or their crosses. These results are consistent with Bulgarian sheep [38], Montenegrin local sheep [44] and Greek Lesbos sheep [47] or with Alpine, European and Middle Eastern breeds [6].

Genetic structure analyses provide information about the differences between breeds, to determine ancestral, pure and hybrid populations [11,44]. The results of the structural analyses showed two gene pools. The first pool consisted of the CVA and SWI breeds, which were placed in a separate gene pool. The likely reason for this is the common ancestor, but also the geographical isolation and relatively small size of the populations, as well as the fact that they are maintained as a genetic resource. However, the second pool consisted of IVA, SUM, SVA, POG, UHR, UK and TUR with a high level of overlap, suggesting that these breeds share a common ancestor and are not clearly differentiated. Regarding genetic differentiation, the highest number of migratory individuals (Nm) (highest Nm value) was found between the Tsurcana breed (Romania) and the Wallachian sheep (Czech Republic) and between the Tsurcana breed (Romania) and the Podgorska sheep (Poland), which is supported by the fact that these breeds had the lowest Fst value of all pairwise comparisons. Although they use different breeding practices, this may indicate that the breeds share a common history. In essence, genetic distance estimates and cluster analysis revealed that a significant factor in population-level genetic differences is geographical distribution, and gene flow is likely to be common and frequent between populations that come into contact in the same or nearby geographical locations. As a result, despite significant phenotypic differences, populations within a given territory often show close genetic relationships [42].

## 5. Conclusions

The selected microsatellite markers are suitable for analyzing genetic diversity in nine selected Central European sheep breeds. The current study of genetic diversity within and among populations showed a high level of polymorphism. Structural analysis revealed the existence of two main pools of breeds: the first group consisted of CVA and SWI. The remaining sheep breeds had a high level of admixture, which may be attributed to common ancestors, the lack of artificial selection pressure, and a high level of gene flow among breeds, as is typical of traditional breeding systems. However, the genetic characteristics of local breeds can be exploited in future breeding strategies to improve production as well as to protect biological diversity and appreciate their cultural significance.

## Figures and Tables

**Table 1:** Statistics over population for each locus: number of identified alleles (K), mean number of alleles per locus over population (Na), mean number of effective alleles per locus over population (Ne), polymorphic information content (PIC); heterozygosity over population: observed (Ho) and expected (He), total expected heterozygosity (Ht), Shannon’s information index (I), fixation index (Fis, Fit, Fst), genetic diversity among population at each locus (Gst), inbreeding coefficient within individuals for each locus (G_IS_), standard deviation (SE), Hardy–Weinberg equilibrium (HW), ns = not significant, *** *p* < 0.001.

Locus	K	HW	Na	Ne	PIC	Ho	He	Ht	Fis	Fit	Fst	I	Gst	Gis
SRCSP23	18	***	12.667	7.782	0.907	0.831	0.856	0.913	0.029	0.09	0.063	2.210	0.050	0.044
SPS113	11	ns	6.647	3.298	0.687	0.712	0.683	0.720	−0.043	0.011	0.052	1.371	0.040	−0.029
TGL53	13	***	10.333	6.248	0.876	0.824	0.825	0.880	0.002	0.064	0.062	1.993	0.050	0.016
INRA23	15	***	9.667	5.628	0.863	0.804	0.789	0.860	−0.019	0.066	0.083	1.870	0.071	−0.004
SRCSP9	13	***	7.444	2.963	0.641	0.756	0.650	0.684	−0.162	−0.105	0.048	1.371	0.038	−0.147
MAF65	14	ns	8.222	4.456	0.782	0.824	0.770	0.805	−0.070	−0.023	0.043	1.700	0.032	−0.055
MCM527	12	ns	7.000	4.268	0.791	0.771	0.754	0.810	−0.023	0.047	0.069	1.579	0.057	−0.009
CSRD247	22	***	11.444	6.053	0.864	0.809	0.810	0.870	0.001	0.069	0.068	1.953	0.056	0.016
ILST11	10	***	8.000	4.953	0.820	0.837	0.788	0.836	−0.062	−0.002	0.057	1.744	0.045	−0.048
OARFCB20	14	ns	9.000	5.095	0.824	0.776	0.771	0.845	−0.006	0.082	0.087	1.754	0.075	0.010
SRCSP5	8	***	5.222	2.836	0.648	0.480	0.635	0.680	0.244	0.294	0.066	1.241	0.050	0.258
INRA63	20	ns	10.000	5.061	0.825	0.802	0.784	0.846	−0.023	0.051	0.073	1.821	0.061	−0.008
SRCSP8	18	***	9.222	3.185	0.687	0.712	0.666	0.703	−0.069	−0.013	0.052	1.506	0.040	−0.046
Mean	14.54		8.883	4.756	0.786	0.765	0.753	0.804	−0.015	0.048	0.063	1.701	0.052	−0.001

**Table 2:** Mean and standard deviation (SE) over loci for each sheep population based on the analysis of the 13 microsatellite markers: number of alleles (N), mean number of different alleles (Na), mean number of effective alleles (Ne), expected heterozygosity (He), observed heterozygosity (Ho), Shannon’s information index (I), inbreeding coefficient (Fis), polymorphic information content (PIC); private allele (PA).

Breed	N	Na	Ne	Ho	He	I	Fis	PIC	PA
CVA	94	7.231	4.248	0.747	0.729	1.559	−0.026	0.697	-
IVA	141	10.84	5.916	0.817	0.797	1.915	−0.02	0.775	4
SUM	118	9.077	5.057	0.769	0.771	1.775	0.006	0.745	1
SVA	122	9.385	4.846	0.778	0.767	1.722	−0.01	0.736	3
POG	130	10.00	5.322	0.771	0.779	1.815	0.015	0.752	5
SWI	92	7.077	3.158	0.710	0.649	1.380	−0.09	0.615	2
UHR	85	6.538	3.941	0.773	0.719	1.517	−0.06	0.683	1
UKR	104	8.000	4.588	0.745	0.767	1.712	0.028	0.737	-
TUR	148	11.38	5.727	0.770	0.795	1.914	0.031	0.776	8
Mean		8.838	4.756	0.765	0.753	1.701	−0.016		

Czech Wallachian sheep (CVA), Improved Wallachian (IVA), Sumava sheep (SUM), Slovakia population: Slovak Wallachian sheep (SVA), Poland population: sheep Polish Mountain sheep (POG), Świniarka (SWI), Uhruska (UHR), Ukraine population: Ukraine sheep (UKR) and Romanian sheep population: Tsurcana (TUR).

**Table 3:** Pairwise genetic differentiation (Fst) (below diagonal) and number of migrants per generation (Nm) (above diagonal). Maximum and minimum observed values are in bold.

Breed	CVA	IVA	SUM	SVA	POG	SWI	UHR	UKR	TUR
CVA	0.000	7.667	5.680	12.273	6.276	3.448	4.412	5.666	6.854
IVA	0.032	0.000	14.856	13.420	18.058	4.752	7.017	10584	21.854
SUM	0.042	0.017	0.000	8.674	9.918	4.071	6.461	7.374	11.094
SVA	0.020	0.018	0.028	0.000	9.494	4.222	6.437	7.360	12.143
POG	0.038	0.014	0.025	0.026	0.000	3.607	5.495	11.419	20.946
SWI	0.068	0.050	0.058	0.056	0.065	0.000	3.127	3.041	4.321
UHR	0.054	0.034	0.037	0.037	0.044	0.074	0.000	5.113	5.940
UKR	0.042	0.023	0.033	0.033	0.021	0.076	0.047	0.000	11.438
TUR	0.035	0.011	0.022	0.020	0.012	0.055	0.040	0.021	0.000

Czech Wallachian sheep (CVA), Improved Wallachian (IVA), Sumava sheep (SUM), Slovakian population: Slovak Wallachian sheep (SVA), Polish population: Polish Mountain sheep (POG), Świniarka (SWI), Uhruska (UHR), Ukrainian population: Ukraine sheep (UKR) and Romanian sheep population: Tsurcana (TUR).

**Table 4:** Genetic distances between sheep populations: Nei’s standard (DS) [25], above the diagonal, and Roger’s distance (DR) [26] below the diagonal.

Breed	CVA	POG	SUM	SVA	SWI	TUR	UHR	UKR	IVA
CVA	0	0.277	0.299	0.125	0.377	0.257	0.362	0.305	0.230
POG	0.229	0	0.192	0.198	0.403	0.095	0.318	0.164	0.108
SUM	0.248	0.194	0	0.212	0.346	0.178	0.264	0.258	0.129
SVA	0.167	0.196	0.201	0	0.317	0.156	0.256	0.255	0.143
SWI	0.307	0.310	0.281	0.282	0	0.323	0.445	0.493	0.288
TUR	0.227	0.133	0.182	0.175	0.287	0	0.297	0.171	0.094
UHR	0.273	0.251	0.229	0.234	0.315	0.244	0	0.333	0.249
UKR	0.248	0.180	0.221	0.223	0.332	0.183	0.257	0	0.186
ZVA	0.211	0.140	0.158	0.163	0.272	0.131	0.222	0.188	0

Czech Wallachian sheep (CVA), Improved Wallachian (IVA), Sumava sheep (SUM), Slovakian population: Slovak Wallachian sheep (SVA), Polish population: Polish Mountain sheep (POG), Świniarka (SWI), Uhruska (UHR), Ukrainian population: Ukraine sheep (UKR) and Romanian sheep population: Tsurcana (TUR).

**Table 5:** Genetic distances between sheep populations, Cavalli–Sforza and Edwards’ distance (1967, DC) is below the diagonal and the distance according to [28] above the diagonal.

Breed	CVA	POG	SUM	SVA	SWI	TUR	UHR	UKR	IVA
CVA	0	0.073	0.079	0.038	0.125	0.066	0.102	0.081	0.061
POG	0.412	0	0.048	0.050	0.123	0.024	0.085	0.042	0.027
SUM	0.417	0.331	0	0.054	0.112	0.043	0.075	0.064	0.033
SVA	0.289	0.341	0.342	0	0.106	0.039	0.073	0.064	0.036
SWI	0.446	0.488	0.451	0.430	0	0.104	0.144	0.142	0.096
TUR	0.390	0.265	0.306	0.317	0.446	0	0.078	0.042	0.022
UHR	0.447	0.461	0.396	0.396	0.479	0.428	0	0.090	0.068
UKR	0.413	0.335	0.376	0.381	0.498	0.322	0.458	0	0.046
IVA	0.363	0.291	0.279	0.290	0.442	0.263	0.407	0.349	0

Czech Wallachian sheep (CVA), Improved Wallachian (IVA), Sumava sheep (SUM), Slovakian population: Slovak Wallachian sheep (SVA), Polish population: Polish Mountain sheep (POG), Świniarka (SWI), Uhruska (UHR), Ukrainian population: Ukraine sheep (UKR) and Romanian sheep population: Tsurcana (TUR).

## Data Availability

The data presented in present study are available in the article and Supplementary Materials.

## Supplementary Materials

The following supporting information can be downloaded at: https://www.mdpi.com/article/10.3390/ani15162400/s1, Table S1: Characterization of 13 microsatellite markers which are recommended by the ISAG and FAO; Table S2: Summary of number alleles per locus (Na); heterozygosity: observed (Ho) and expected (He); Chi-Square Tests for Hardy–Weinberg Equilibrium; polymorphic information content (PIC); Table S3: Analysis of molecular variance (AMOVA) and F-statistic of nine sheep breeds based on genotyping of 13 microsatellite markers; Table S4: Estimated posterior probabilities Mean LnP(K) for a different number of inferred clusters (K) and Δ K.
