# Supplementary material for: Genetic Diversity and Population Structure of Nine Local Sheep Populations Bred in the Carpathia Area of Central Europe Revealed by Microsatellite Analysis
Source: Animals (Basel). 2025 Aug 15;15(16):2400. doi: 10.3390/ani15162400 (PMC12383097; doi:10.3390/ani15162400)
Supplement: Supplementary file 1 [file animals-15-02400-s001.zip › animals-3787310-supplementary.pdf]

Supplementary

Table S1. Characterization of 13 microsatellite markers which are recommended by the ISAG and FAO.

Microsatellite marker	Chr. No	Primer sequence 5´- 3´	Deys	Product size bp	Annealing temperature (°C)
Multiplex 1					
SRCRSP9	12	F: AGAGGATCTGGAAATGGAATC<br>R: GCACTCTTTTCAGCCCTAATG	FAM	99-156	55
MAF65	OAR15	F: AAAGGCCAGAGTATGCAATTAGGAG<br>R: CCACTCCTCCTGAGAATATAACATG	VIC	115-160	55
MCM527	5	F: GTCCATTGCCTCAAATCAATTC<br>R: AAACCACTTGACTACTCCCCAA	PET	160-190	55
ETH10	15	F: GTTCAGGACTGGCCCTGCTAACA<br>R: CCTCCAGCCCACTTTCTCTTCTC	VIC	200-230	55
CSRD247		F: GGACTTGCCAGAACTCTGCAAT<br>R: CACTGTGGTTTGTATTAGTCAGG	PET	220-250	55
ILSTS11	BTA14	F: GCTTGCTACATGGAAAGTGC<br>R: CTAAAATGCAGAGCCCTACC	FAM	250-300	55
Multiplex2					
SRCRSP23	UNK	F: TGAACGGGTAAAGATGTG<br>R: TGTTTTTAATGGCTGAGTAG	FAM	80-120	55
SPS113	BTA10	F: CCTCCACACAGGCTTCTCTGACTT<br>R: CCTAACTTGCTTGAGTTATTGCCC	PET	130-160	55
TGLA53(D1653)	16	F: GCTTTCAGAAATAGTTTGCATTCA<br>R: ATCTTCACATGATATTACAGCAGA	VIC	130-190	55
INRA23	3	F: GTAGAGCTACAAGATAAACTTC<br>R: TAACTACAGGGTGTTAGATGAACT	FAM	190-220	55
Multiplex3					
OarFCB20	11	F: AAATGTGTTTAAGATTCCATACAGTG<br>R: GGAAAACCCCCATATATACCTATAC	NED	90-120	55
SRCRSP5	18	F: GGACTCTACCAACTGAGCTACAAG<br>R: TGAAATGAAGCTAAAGCAATGC	FAM	120-180	55
INRA063 (D1855)	18	F: ATTTGCACAAGCTAAATCTAACC<br>R: AAACCACAGAAATGCTTGGAAG	VIC	160-190	55
SRCRSP8	UNK	F: TGCGGTCTGGTTCTGATTTCAC<br>R: CTGCATGAGAAAGTCGATGCTTAG	FAM	210-260	55

Table S2. Summary of number alleles per locus (Na), heterozygosity: observed (Ho) and expected (He), Chi-Square Tests for Hardy-Weinberg Equilibrium, Polymorphic Information Content (PIC) in all studied microsatellite markers by breed

**Breed**		**1**	**2**	**3**	**4**	**5**	**6**	**7**	**8**	**9**	**10**	**11**	**12**	**13**
CVA	Na	12	6	7	8	4	9	5	7	8	9	6	6	7
	Ho	0.771	0.629	0.882	0.781	0.600	0.857	0.771	0.829	0.743	0.812	0.429	0.771	0.824
	He	0.903	0.607	0.779	0.747	0.528	0.796	0.692	0.753	0.728	0.808	0.653	0.749	0.740
	HWE	ns*	***	ns	**	ns	ns	ns	ns	ns	ns	***	ns	ns
	PIC	0.894	0.543	0.766	0.754	0.492	0.772	0.629	0.716	0.683	0.789	0.593	0.709	0.719
IVA	Na	15	8	12	13	7	7	10	17	9	12	7	13	11
	Ho	0.864	0.712	0.915	0.932	0.661	0.864	0.831	0.864	0.898	0.915	0.559	0.932	0.678
	He	0.891	0.691	0.878	0.851	0.636	0.785	0.817	0.896	0.848	0.859	0.695	0.863	0.648
	HWE	ns	ns	ns	ns	ns	ns	ns	ns	ns	ns	***	ns	ns
	PIC	0.881	0.647	0.866	0.835	0.608	0.754	0.793	0.887	0.830	0.843	0.658	0.851	0.617
SUM	Na	14	8	11	10	8	7	5	11	9	9	6	11	9
	Ho	0.870	0.826	0.826	0.932	0.761	0.870	0.761	0.804	0.826	0.739	0.435	0.761	0.587
	He	0.889	0.774	0.865	0.874	0.647	0.784	0.780	0.815	0.804	0.732	0.621	0.816	0.625
	HWE	ns	**	ns	*	ns	ns	ns	ns	ns	ns	***	ns	ns
	PIC	0.879	0.741	0.850	0.872	0.591	0.752	0.745	0.792	0.7823	0.692	0.582	0.780	0.603
SVA	Na	14	5	12	10	8	11	8	12	8	10	4	11	9
	Ho	0.929	0.625	0.821	0.741	0.768	0.821	0.750	0.786	0.857	0.714	0.643	0.875	0.786
	He	0.882	0.668	0.888	0.838	0.664	0.779	0.759	0.817	0.766	0.713	0.674	0.793	0.724
	HWE	ns	ns	ns	**	ns	ns	ns	***	ns	ns	ns	ns	ns
	PIC	0.871	0.601	0.877	0.832	0.625	0.753	0.722	0.794	0.735	0.671	0.621	0.764	0.696
POG	Na	14	8	11	12	8	7	9	15	10	10	4	12	10
	Ho	0.809	0.723	0.681	0.979	0.660	0.745	0.872	0.936	0.936	0.809	0.340	0.723	0.809
	He	0.888	0.690	0.758	0.876	0.586	0.781	0.784	0.873	0.848	0.860	0.638	0.804	0.739
	HWE	ns	ns	ns	ns	ns	ns	ns	ns	ns	**	***	ns	ns
	PIC	0.878	0.643	0.734	0.863	0.55	0.748	0.754	0.859	0.829	0.845	0.585	0.777	0.712
SWI	Na	10	7	8	6	9	7	5	9	6	6	4	8	7
	Ho	0.882	0.941	0.618	0.529	1.000	0.824	0.618	0.794	0.794	0.559	0.471	0.706	0.500
	He	0.805	0.782	0.776	0.554	0.721	0.663	0.640	0.716	0.702	0.526	0.513	0.599	0.434
	HWE	ns	ns	***	**	**	ns	***	ns	ns	ns	***	*	ns
	PIC	0.78	0.749	0.741	0.530	0.681	0.630	0.5873	0.686	0.670	0.493	0.468	0.572	0.407
UHR	Na	9	5	11	5	6	9	6	6	6	6	4	5	7
	Ho	0.579	0.526	0.947	0.737	1.000	0.947	0.737	0.842	0.895	0.895	0.368	0.789	0.737
	He	0.715	0.586	0.856	0.681	0.729	0.805	0.752	0.755	0.807	0.756	0.514	0.766	0.627
	HWE	***	ns	ns	ns	ns	ns	ns	**	*	ns	***	ns	***
	PIC	0.696	0.531	0.841	0.635	0.685	0.780	0.710	0.715	0.779	0.723	0.467	0.728	0.585
UKR	Na	10	5	8	10	7	7	7	9	7	8	5	11	10
	Ho	0.895	0.737	0.842	0.842	0.684	0.789	0.737	0.632	0.737	0.632	0.526	0.895	0.737
	He	0.837	0.625	0.758	0.837	0.733	0.759	0.734	0.765	0.803	0.827	0.701	0.846	0.751
	HWE	ns	ns	ns	ns	ns	ns	ns	ns	ns	*	*	ns	ns
	PIC	0.820	0.568	0.727	0.8187	0.698	0.728	0.692	0.737	0.775	0.804	0.650	0.829	0.729
TUR	Na	16	8	13	13	10	10	8	17	9	11	7	13	13
	Ho	0.879	0.690	0.879	0.759	0.667	0.700	0.867	0.797	0.850	0.900	0.550	0.767	0.700
	He	0.894	0.720	0.867	0.841	0.611	0.783	0.828	0.903	0.789	0.861	0.709	0.823	0.705
	HWE	ns	ns	ns	***	ns	ns	ns	ns	ns	ns	*	ns	***
	PIC	0.891	0.699	0.863	0.836	0.576	0.750	0.805	0.898	0.760	0.846	0.672	0.802	0.685

Czech sheep population: CVA Czech Wallachian sheep, IVA Improved Wallachian, SUM Sumava sheep; Slovakia population: SVA Slovak Wallachian sheep; Poland population: POG Polish Mountain sheep, SWI Swiniarka, UHR Uhruska; Ukraine population: UKR Ukraine sheep; and Romanian sheep population: TUR-Tsurcana; 1-SRCSP23,2- SPS113, 3-TGL53; 4-INRA23, 5-SRCSP9, 6-MAF65, 7-MCM527, 8-CSRD247, 9-ILST11, 10-OARFCB20, 11-SRCSP5, 12-INRA63; 13-SRCSP8; ns*-not signifiant; *p< 0.05, **p <0.01,***p<0.001.

Table S3. Analysis of molecular variance (AMOVA) and F- statistic of nine sheep breeds based on genotyping of 13 microsatellite markers.

Source	Df	SS	MS	Est. Var.	%
Among Populations	8	210.897	26.362	0.260	5%
Among Individuals	366	1848.709	5.051	0.034	1%
Within Individuals	375	1869.000	4.984	4.984	94%
Total	749	3928.607		5.277	100%

Table S4. Estimated posterior probabilities Mean LnP(K) for different number of inferred clusters (K) and Δ K.

K	Mean LnP(K)	Δ K
1	-19572.36000	NA
2	-19031.67500	103.43010
3	-18720.18500	0.75723
4	-18452.10500	1.40087
5	-18311.41500	0.43881
6	-18113.36500	1.92715
7	-18214.95000	0.72029
8	-18249.78000	0.88712
9	-18205.72500	0.33929
